# Comparison of Waist Circumference and Waist-to-Hip Ratio in Assessing Abdominal Obesity and Cardiometabolic Risk Among Women Aged 15-49 Years in India: Evidence From a National Cross-Sectional Survey

**DOI:** 10.7759/cureus.109516

**Published:** 2026-05-23

**Authors:** Priyanshu Sharma, Mamta Chauhan

**Affiliations:** 1 Public Health, Institute of Health Management Research (IIHMR) University, Jaipur, IND

**Keywords:** abdominal obesity, diabetes, hypertension, india, waist circumference, waist-to-hip ratio, women

## Abstract

Introduction

Abdominal obesity (AO) has become a major public health challenge, particularly in South Asian populations where risk occurs at lower body mass index (BMI) levels. Waist circumference (WC) and waist-to-hip ratio (WHR) are widely used measures of AO, yet their comparative performance in identifying individuals at risk remains unclear. This study aims to compare WC and WHR in estimating AO, examine their agreement and discordance, and assess their association with diabetes and hypertension among women in India.

Methods

We utilised the data from the fifth round of the National Family Health Survey, 2019-21, a nationally representative cross-sectional survey conducted across all 36 states and union territories of India. The survey collected information from 724,115 women aged 15-49 years. Women who were pregnant at the time of the survey, observations with extreme anthropometric values, and missing values were excluded from the study. The final analytical sample comprised 664,646 women. AO was defined using WC >80 cm and WHR ≥0.85. Agreement between measures was assessed using percentage agreement and Cohen’s kappa statistic. Discordance analysis was conducted to compare socioeconomic and behavioural profiles across classification groups. Multivariate logistic regression models were used to examine associations of WC- and WHR-defined AO with diabetes and hypertension after adjusting for socioeconomic and behavioural covariates.

Results

The prevalence of AO among women was 39.9% using WC and 56.6% using WHR. Agreement between the two measures was fair (66.8%; kappa=0.36), with lower agreement among younger women. Nearly one-fourth of women (24.3%) were classified as abdominally obese by WHR alone compared to 7.6% by WC alone, indicating high discordance between the two measures. Women identified through WC had a higher prevalence of overweight/obesity, urban residence, higher wealth status, and parity, whereas the WHR-only group was more similar to the normal population. WC-defined AO also showed stronger alignment with BMI, while WHR classified a substantial proportion of underweight and normal BMI women as abdominally obese. WC showed a stronger association with cardiometabolic outcomes, with higher odds of diabetes (adjusted odds ratio (AOR)=1.73) and hypertension (AOR=1.51) compared to WHR (AOR=1.33 for both outcomes).

Conclusions

WC and WHR identify different population groups and are not interchangeable measures of AO. While WHR yields higher prevalence estimates, WC demonstrates stronger alignment with BMI and cardiometabolic risk. Given its simplicity, interpretability, and stronger predictive value, WC may be a more appropriate tool for population-level screening and risk identification among women in India.

## Introduction

Obesity is now widely recognised as a major health challenge of the 21st century. The burden is increasing in low- and middle-income countries [1]. However, health risks linked to obesity are not determined only by the amount of body fat. The distribution of fat in the body plays an equally important role in determining risk. Abdominal or central obesity is considered the most dangerous form of obesity and is more strongly linked to adverse cardiometabolic outcomes such as diabetes, hypertension, cardiovascular diseases and certain types of cancers [2-5].

This distinction matters even more in South Asian populations. Evidence shows that South Asians have higher levels of abdominal obesity (AO) even at lower body mass index (BMI) levels, putting them at higher risk despite appearing non-obese [6]. As a result, depending on BMI alone may underestimate the risk. There is a growing need to better understand and measure AO in both research and public health practice. The burden of AO has increased substantially over time. A systematic review was conducted in 2020 involving 13.2 million individuals globally. The AO prevalence was estimated to be 41.5%, indicating that approximately two in five adults are affected worldwide [7]. If the present trend continues, this burden is likely to increase further, underscoring the need for early identification and prevention efforts.

Waist circumference (WC) and waist-to-hip ratio (WHR) are the most commonly used measures of AO due to their simplicity, low cost and feasibility in large-scale surveys [8]. However, they capture different aspects of fat distribution in the body. WC indicates the absolute accumulation of abdominal fat, whereas WHR captures the proportion of WC to hip circumference, thereby informing relative fat distribution rather than absolute abdominal fat mass. This raises an important question - do these measures identify the same individuals at risk?

The existing literature on WC and WHR provides mixed answers. Some studies suggest that WC is a stronger predictor of cardiometabolic outcomes as it is more closely linked to visceral fat [8-10]. Others have argued that WHR may better capture cardiovascular risk [11,12]. Most of the work, however, has focused on how strongly these measures are associated with the outcome. Limited attention has been given to how they differ in classifying individuals or understanding the extent of agreement between these two important indicators. This gap is important as it will have direct implications on screening, programme strategies and estimation of disease burden.

India is undergoing an epidemiological and nutritional transition, with increasing levels of overweight, obesity and non-communicable diseases [12]. There is growing recognition of AO. However, the evidence remains limited and fragmented in India. Most of the previous studies on AO are limited to specific regions or small community and urban samples, thereby restricting generalisability [13-16]. The primary focus of these studies was mainly to assess cardiometabolic risks, and AO was often included as a covariate. A few large-scale studies have estimated the prevalence of AO and examined its determinants, but they have largely remained descriptive [17-19]. Much less has been done to systematically examine the relationship between the WC and WHR. As a result, there is limited understanding of how these measures differ in identifying individuals and capturing risk patterns within the Indian population.

The inclusion of WC and hip circumference measurements in the fifth round of the National Family Health Survey (NFHS-5) conducted in 2019-21 provides an opportunity to study these indicators in greater detail. The NFHS-5 reports that nearly 40% of women and 12% of men aged 15-49 years in India are abdominally obese [20], affecting a substantial proportion of women. Women in the reproductive age group experience distinct biological and life-course transitions, including pregnancy, childbirth and hormonal changes, which influence fat distribution and metabolic risk [21]. Whether WC and WHR capture these dynamics in similar or different ways largely remains unexplored by previous studies.

In this study, we provide a detailed comparison of WC and WHR in identifying AO among women aged 15-49 years in India. This study goes beyond simple prevalence estimates to examine the level of agreement between the two measures, the extent and patterns of discordance in classification, and the socio-economic and behavioural characteristics of groups identified by each measure. We also examined the relationship of AO, as defined by WC and WHR, with key health outcomes - diabetes and hypertension. By doing so, the study generated evidence on whether these measures can be used interchangeably and which may be more appropriate for population-level assessment and risk identification in the Indian context.

## Materials and methods

Data source and study population

This study utilises data from the NFHS-5. NFHS-5 is a nationally representative cross-sectional household survey, conducted across all 36 states and union territories of India during 2019-21 under the stewardship of the Ministry of Health and Family Welfare, Government of India [20]. NFHS-5 is a part of the global Demographic and Health Surveys (DHS) programme. The anonymised dataset was accessed through the DHS Program website: https://dhsprogram.com.

NFHS-5 covered 636,699 households and collected information from 724,115 women aged 15-49 years. The survey employed a stratified two-stage sampling design and collected detailed information on demographic and socio-economic characteristics, health outcomes and anthropometric measurements. It also included measurement of WC and hip circumference for all eligible women aged 15-49 years.

Exclusion criteria

Women who were pregnant at the time of the survey were excluded from the analysis. Outliers were identified using the mean ±3 standard deviation criteria. It is a common statistical approach based on the empirical 3-sigma rule, where approximately 99.7% of observations in a normal distribution fall within this range. The values beyond this range were treated as extreme observations and excluded from the analysis. The observations with missing values were also excluded from the analysis. The final analytical sample comprised approximately 664,646 women.

Measures of AO

The primary outcome was AO, assessed using two commonly used indicators: WC and WHR. AO was defined using standard cut-offs recommended for women: WC >80 cm and WHR ≥0.85.

WC was measured at the midpoint between the lower margin of the last palpable rib and the top of the iliac crest. Hip circumference was measured at the widest portion of the hips. WHR was calculated as the ratio of WC to hip circumference. In this study, AO was examined separately using WC and WHR to compare their classification and association with cardiometabolic risk outcomes.

Covariates

A range of socio-demographic, reproductive and behavioural variables were included in the analysis. These included age (15-34 and 35-49 years), place of residence (urban/rural), education level, wealth quintile, religion, caste, parity (number of children ever born), diet patterns (vegetarian/non-vegetarian), junk food consumption (categorized as low/moderate vs high), alcohol use, tobacco use and type of occupation (not working, sedentary, or physically active).

BMI was calculated as weight in kilograms divided by height in metres squared and categorised as underweight (<18.5 kg/m^2^), normal (18.5-24.9 kg/m^2^), overweight (25.0-29.9 kg/m^2^) and obese (≥30.0 kg/m^2^).

In addition, two cardiometabolic outcomes were included to assess the association with AO.

Hypertension

Women were classified as having hypertension if they had a systolic blood pressure greater than or equal to 140 mmHg, a diastolic blood pressure greater than or equal to 90 mmHg, or were currently taking antihypertensive medication to lower their blood pressure.

Diabetes

Women were classified as having diabetes (high blood glucose) if they had a random blood glucose level greater than 140 mg/dL or were currently taking medication to lower their glucose level.

Statistical analysis

Descriptive statistics were used to estimate the distribution of women across the four categories of AO. Age-stratified analysis was conducted to examine variations across life stages. Agreement between WC and WHR in classifying AO was assessed using percentage agreement and Cohen’s kappa statistic.

The socio-economic and behavioural profiles of the four groups were compared using proportions. To examine the relationship between AO and BMI, the prevalence of AO (defined separately using WC and WHR) was estimated across BMI categories. Logistic regression models were then used to assess the association between BMI categories and AO for each measure.

To evaluate the relationship with cardiometabolic outcomes, separate multivariate logistic regression models were estimated for diabetes and hypertension. AO (defined using WC and WHR separately) was included as the key independent variable, adjusting for socio-demographic and behavioural covariates. Adjusted odds ratios (AORs) with 95% confidence intervals were reported.

All analyses accounted for the complex survey design using appropriate sampling weights, clustering, and stratification to ensure nationally representative estimates. Statistical analyses were performed using Stata version 16 (StataCorp LLC, College Station, TX, US) [22].

## Results

**Figure 1:**
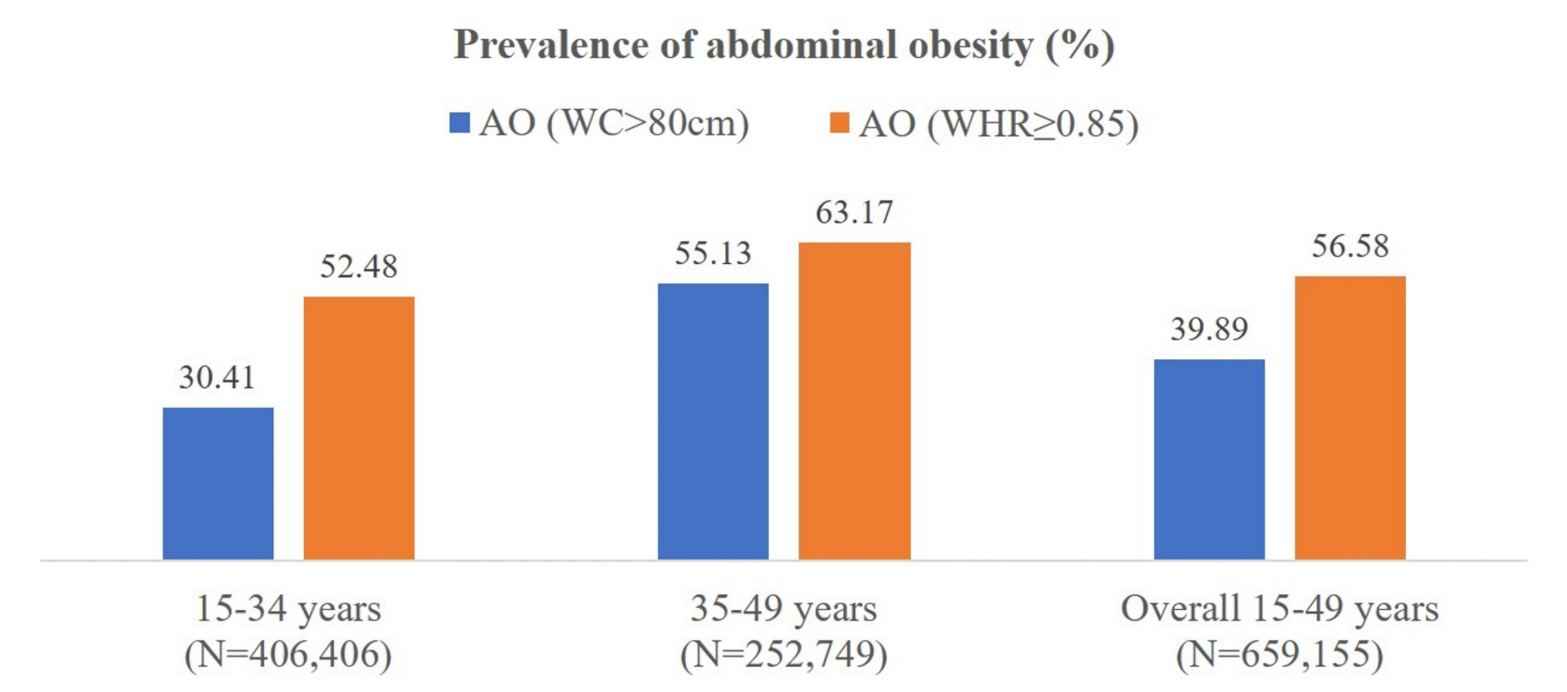
Prevalence of abdominal obesity (AO) across age groups by both measures (WC and WHR) among women aged 15-49 years, 2019-21 WC: waist circumference; WHR: waist-to-hip ratio

AO is widely prevalent among women aged 15-49 years in India, with substantial variation depending on the type of measure used. Overall, the prevalence was estimated at 39.89% using WC and 56.58% using WHR (Figure 1). This reflects an overwhelming burden on women in the reproductive age group. We compared WC and WHR in identifying AO among women across age groups. Among younger women aged 15-34 years, the prevalence of AO was 30.41% using WC and 52.48% using WHR. In contrast, among older women aged 35-49 years, the prevalence increased sharply to 55.13% using WC and 63.17% using WHR. This indicates a clear age-related increase in AO for both measures, with a steeper rise observed for WC compared to WHR.

Classification and discordance between WC and WHR

To better understand these differences in AO prevalence, women were classified into four groups based on WC and WHR. These were (a) normal by both measures, (b) abdominally obese by both measures, (c) WC-only, and (d) WHR-only. The distribution across these groups showed clear differences (Table 1). Only 35.81% of women were normal by both measures. A smaller share (7.62%) was identified as obese only by WC, whereas a much larger share (24.31%) was identified as obese only by WHR.

**Table 1:** Proportion of women aged 15-49 years by category of AO classification, 2019-21 AO: abdominal obesity; WC: waist circumference; WHR: waist-to-hip ratio

S. No.	Category	15-34 years, n (%) (N=406,406)	35-49 years, n (%) (N=252,749)	Total 15-49 years, n (%) (N=659,155)
1	Normal	169,925 (41.81)	66,088 (26.15)	236,013 (35.81)
2	AO by both	100,368 (24.70)	112,354 (44.45)	212,722 (32.27)
3	AO by WC only	23,211 (5.71)	26,998 (10.68)	50,208 (7.62)
4	AO by WHR only	112,902 (27.78)	47, 309 (18.72)	160,212 (24.31)

Age-specific classification further highlights this divergence. Among younger women (15-34 years), a larger proportion was classified as WHR-only (27.78%) compared to WC-only (5.71%), indicating that WHR identifies a broader group in early adulthood. In contrast, among older women (35-49 years), the proportion identified by both measures increases substantially (44.45%), and the gap between the WC-only and WHR-only group narrows. This shows that WHR captures a much larger group of women (particularly at younger ages) than WC does.

Agreement between WC and WHR

The overall agreement between the two measures was 66.8%, which means that both WC and WHR classified about two-thirds of women in the same way, either as abdominally obese or non-obese. The expected agreement (48.1%) represents the proportion of agreement that would be anticipated by chance alone based on the distribution of classifications by the two measures. Cohen’s kappa adjusted for this chance agreement and estimated the agreement beyond chance. The kappa statistic ranges from 0 to 1, where 0 indicates no agreement beyond chance and 1 indicates perfect agreement. The kappa value was only 0.36, indicating a fair agreement beyond chance (Table 2). It suggests that the two measures differed in classifying a substantial proportion of women and therefore may not be used interchangeably.

**Table 2:** Agreement between WC and WHR in identifying abdominal obesity among women aged 15-49 years, 2019-21 WC: waist circumference; WHR: waist-to-hip ratio

Age group	Agreement	Expected Agreement	Kappa	Std. Err.	Z	p-value
Overall 15-49 years	66.81%	48.12%	0.3603	0.0011	318.21	<0.001
15-34 years	64.97%	48.39%	0.3212	0.0014	235.96	<0.001
35-49 years	69.79%	50.73%	0.3869	0.0019	200.37	<0.001

Age-stratified analysis showed slightly lower agreement among younger women (64.9%, kappa=0.32) and slightly higher agreement among older women (69.8%, kappa=0.39), suggesting greater differences between the two measures in younger women. The smaller values of standard error indicate the greater reliability of the estimate. High Z values and p-values below 0.001 indicate that the agreement observed between WC and WHR was statistically significant across all age groups.

Socioeconomic and behavioural profile of discordant groups

Table 3 presents the socioeconomic, demographic, and behavioural characteristics of women across different categories of AO classification. This table helps in understanding the profile of these four groups and assessing how similar or different they are based on various characteristics. This comparison is important because it provides insight into whether WC and WHR identify similar or distinct population subgroups. The findings show meaningful differences across four groups.

**Table 3:** Distribution of socioeconomic, demographic and behavioural characteristics across categories of AO classification among women (%), 2019-21 AO: abdominal obesity; WC: waist circumference; WHR: waist-to-hip ratio

S. No.	Covariates	Overall Prevalence, n (%)	Prevalence by AO Categories (%)			
			Normal, n (%)	AO by both, n (%)	AO by WC only, n (%)	AO by WHR only, n (%)
1	Urban residence	210,649 (31.96)	62,561 (26.51)	83,043 (39.04)	22,039 (43.9)	43,005 (26.84)
2	Overweight/obese	158,203 (24.01)	13,417 (5.69)	103,362 (48.61)	33,304 (66.38)	8,121 (5.07)
3	10+ years of education	265,328 (40.25)	95,590 (40.5)	86,399 (40.62)	22,335 (44.48)	61,004 (38.08)
4	Rich	266,577 (40.44)	80,198 (33.98)	106,955 (50.28)	29,681 (59.12)	49,743 (31.05)
5	Religion					
	Hindu	539,321 (81.81)	201,338 (85.3)	166,587 (78.31)	41,652 (82.96)	129,745 (80.98)
	Muslim	86,317 (13.1)	24,423 (10.35)	32,552 (15.3)	5,819 (11.59)	23,524 (14.68)
	Christian	15,542 (2.36)	4,713 (2)	6,056 (2.85)	1,458 (2.9)	3,315 (2.07)
	Sikh	10,055 (1.53)	2,192 (0.93)	5,663 (2.66)	619 (1.23)	1,581 (0.99)
	Buddhist	4,173 (0.63)	1,959 (0.83)	952 (0.45)	387 (0.77)	875 (0.55)
	Jain	1,268 (0.19)	430 (0.18)	378 (0.18)	170 (0.34)	289 (0.18)
	Others/no	2,480 (0.38)	959 (0.41)	534 (0.25)	103 (0.21)	883 (0.55)
6	Caste					
	Schedule caste	144,746 (23.06)	53,244 (23.38)	45,603 (22.69)	9,397 (19.42)	36,501 (24.23)
	Schedule tribe	61,772 (9.84)	26,408 (11.6)	12,529 (6.23)	2,652 (5.48)	20,183 (13.4)
	Other backward class	282,887 (45.06)	105,289 (46.23)	88,808 (44.19)	23,684 (48.94)	65,107 (43.22)
	Other	133,822 (21.32)	41,173 (18.08)	52,510 (26.13)	12,407 (25.64)	27,733 (18.41)
7	Nonvegetarians	352,503 (53.48)	116,690 (49.44)	122,289 (57.49)	28,106 (55.98)	85,418 (53.32)
8	Junk food high score	108,376 (16.44)	36,119 (15.3)	36,755 (17.28)	8,364 (16.66)	27,137 (16.94)
9	Two or more children ever born	372,940 (56.58)	115,540 (48.95)	145,384 (68.34)	35,475 (70.66)	76,541 (47.78)
10	Alcohol use	5,110 (0.78)	1,715 (0.73)	1,364 (0.64)	295 (0.59)	1,736 (1.08)
11	Tobacco use	27,624 (4.19)	10,682 (4.53)	7,971 (3.75)	1,574 (3.14)	7,396 (4.62)
12	Work experience					
	Not working	628,304 (95.77)	224,198 (95.37)	203,050 (96.01)	47,587 (95.5)	153,470 (96.11)
	Sedentary work	8,115 (1.24)	2,860 (1.22)	2,727 (1.29)	726 (1.46)	1,802 (1.13)
	Active - irregular	9,364 (1.43)	4,091 (1.74)	2,445 (1.16)	580 (1.16)	2,249 (1.41)
	Active - regular	10,293 (1.57)	3,934 (1.67)	3,265 (1.54)	938 (1.88)	2,155 (1.35)

Urban residence varied strongly across the groups. Overall, 31.9% of women lived in urban areas. This proportion increased to 39% among women classified as obese by both measures and further to 43.9% among those identified only by WC. This share was lower in the normal group (26.5%) and also relatively low in the WHR-only group.

The differences in BMI across these groups were striking. Overall, 24% of women were overweight or obese as per BMI criteria. However, this proportion was low in the WHR-only group (5.1%) and normal group (5.7%). In comparison, nearly half of the women in both the obese group were overweight or obese (48.6%), which increased further to 66.4% in the WC-only group.

The difference in wealth showed a similar pattern, with higher representation in the both-obese (50.3%) and WC-only (59.1%) groups compared to the normal group (34%) and WHR-only group (31%). Higher educational attainment varied little across groups, with only a marginally higher share in the WC-only category. Variations by religion were modest, whereas caste differences were more distinct. Scheduled Tribe women were more concentrated in the normal and WHR-only groups, while women from ‘Other’ caste groups were overrepresented in the both-obese and WC-only categories.

Dietary patterns showed minimal variation across groups. Parity showed a strong pattern. Women with two or more children were more prevalent in the both-obese (68.3%) and WC-only (70.7%) groups, while the WHR-only group closely resembled the normal group. Behavioural factors such as alcohol and tobacco use showed only minor differences. Similarly, work patterns were largely uniform, with over 95% of women not engaged in formal employment across all groups.

Relationship with BMI

BMI has historically been used as a measure of overall or general obesity. Based on BMI values, women were classified into four categories, i.e. underweight (<18.5 kg/m^2^), normal (18.5-24.9 kg/m^2^), overweight (25.0-29.9 kg/m^2^) and obese (≥30.0 kg/m^2^). Table 4 shows the variation of WC- and WHR-defined AO across these BMI categories. This helped in understanding how closely each AO measure aligns with general obesity measured through BMI. In simple terms, it assesses whether women identified as abdominally obese by WC or WHR also tend to fall into higher BMI categories.

**Table 4:** Prevalence, odds ratios (ORs) and 95% confidence intervals (CIs) for abdominal obesity (AO) defined by WC and WHR across BMI categories among women aged 15-49 years, 2019-21 WC: waist circumference; WHR: waist-to-hip ratio

BMI Category	AO Defined by WC >80 cm			AO Defined by WHR ≥0.85		
	Prevalence, n (%)	OR (95% CI)	p-value	Prevalence, n (%)	OR (95% CI)	p-value
Low (<18.5)	6,513 (5.29)	1.00 (Ref)	-	46,913 (38.14)	1.00 (Ref)	-
Normal (18.5-25)	119,427 (31.69)	7.99 (7.78-8.20)	<0.001	214,024 (56.79)	2.17 (2.14-2.20)	<0.001
Overweight (25-30)	96,846 (83.42)	81.09 (78.72-85.53)	<0.001	80,929 (69.71)	3.96 (3.89-4.03)	<0.001
Obesity (>30)	39,819 (94.56)	265.73 (252.99-279.11)	<0.001	30,553 (72.56)	4.75 (4.62-4.88)	<0.001

The findings show that the relationship between AO and BMI differed substantially between the two measures. WC showed a steep and consistent increase across BMI categories. Only 5.3% of underweight women were classified as abdominally obese using WC, compared to 31.7% of women with normal BMI, 83.4% of overweight women, and 94.5% of obese women. In contrast, WHR identified a substantial proportion of women as abdominally obese even at lower BMI levels. Among underweight women, 38.1% were classified as abdominally obese using WHR, increasing to 56.8% among women with normal BMI and reaching 69.7% and 72.6% among overweight and obese women, respectively. However, the increase across BMI categories was more gradual in WHR compared to WC.

These patterns were further validated in a regression analysis. Women with normal BMI had nearly eight times higher odds of AO when defined using WC, which further increased sharply to 81 times among overweight and 266 times among obese women. On the other hand, the corresponding increase in odds ratios for WHR was much smaller, ranging from approximately 2 to 5 across BMI categories.

Association with diabetes and hypertension

Table 5 examines the association of AO with two major cardiometabolic conditions, i.e. diabetes and hypertension. This comparison is important because it helps us to assess the clinical relevance of WC and WHR in identifying health risk, particularly among Indian women.

**Table 5:** Prevalence of diabetes and hypertension by WC and WHR among women aged 15-49 years, 2019-21

S. No.	Abdominal Obesity (By Measure)	Diabetes, n (%)	Hypertension, n (%)
1	Using waist circumference (WC)		
	Not obese	20,540 (5.18)	53,416 (13.48)
	Obese	31,428 (11.95)	60,755 (23.11)
2	Using waist-to-hip ratio (WHR)		
	Not obese	17,074 (5.97)	39,482 (13.79)
	Obese	34,894 (9.36)	74,688 (20.03)

The prevalence of both diabetes and hypertension was higher among women with AO as compared to non-obese women, irrespective of the measure used. However, the increase from non-obese to obese women was more pronounced when AO was defined using WC. For instance, the prevalence of diabetes increased from 5.2% among women without AO to 12% among women with AO defined using WC. In comparison, when AO was defined using WHR, the prevalence of diabetes increased from 6% to 9.4%. A similar pattern was observed for hypertension.

Multivariate logistic regression analysis further confirmed these findings. Table 6 further evaluates the comparative relevance of WC and WHR by examining their independent association with diabetes and hypertension after adjusting for socioeconomic and behavioural factors. The analysis is important because it helps in assessing whether the observed association remains significant even after accounting for differences in their background characteristics among women. The results showed that WC-defined AO had a stronger association with both diabetes and hypertension compared to WHR.

**Table 6:** Adjusted odds ratios (AORs) and 95% confidence intervals (CIs) for the association of abdominal obesity (WC and WHR) with diabetes and hypertension among women aged 15-49 years, 2019-21 Note: AORs were derived from multivariable logistic regression models adjusted for socio-demographic and behavioural characteristics.

Measurement of Abdominal Obesity	Outcome (AOR, 95% CI)	
	Diabetes	Hypertension
WC	1.73 (1.69-1.76)	1.51 (1.49-1.53)
WHR	1.33 (1.30-1.35)	1.33 (1.31-1.34)

After adjusting for background factors, women with WC-defined AO had 1.73 times higher odds of diabetes, whereas the corresponding odds for WHR-defined AO were 1.33 times higher compared to normal women. A similar pattern was observed for hypertension, where WC-defined obesity showed a stronger association (AOR=1.51) compared to WHR-defined AO (AOR=1.33).

## Discussion

This study highlights a high burden of AO among women aged 15-49 years in India, with prevalence ranging from 39.9% to 56.6% depending on the type of anthropometric measure used. It shows that WHR identifies a substantially higher proportion of women as abdominally obese compared to WC. This gap is wider among younger women. This finding is consistent with the broader understanding that WC and WHR capture different aspects of body shape and fat distribution. The WHO expert consultation (2008) notes that WC and WHR differ in their measurement properties and in their relationship with age, sex, ethnicity, BMI and cardiometabolic risk [8]. From a public health perspective, this has important implications because the choice of anthropometric indicator can considerably alter the estimated burden of AO and influence the identification of populations at risk.

A key contribution of this study is the direct comparison between WC and WHR using a large nationally representative sample of Indian women. The analysis shows high discordance between the two measures, implying that they differ in classifying a substantial proportion of women. Nearly one-fourth of women were identified as abdominally obese by WHR alone, whereas only a small fraction were identified exclusively by WC. This discordance was higher among younger women, where WHR classified a large proportion of women as abdominally obese despite relatively lower levels of overall obesity. This likely reflects the underlying difference between the two measures. WHR captures relative fat distribution, while WC measures the accumulation of absolute abdominal fat.

These findings are similar to the study by Haregu et al. among the urban Kenyan population, which also reported a higher prevalence of AO using WHR compared to WC and demonstrated considerable discordance between anthropometric measures. They observed that WHR generally had lower agreement with other obesity indicators [23]. Another population-based study by Mahmoud and Sulaiman among adults in the United Arab Emirates reported fair-to-moderate agreement between WC and WHR and poor agreement between BMI and WHR across different age and sex groups [24]. These studies suggest that anthropometric indicators should not be used interchangeably because they classify different population subgroups and differ in their association with cardiometabolic risk.

Several other important findings emerge from this study. When women were classified into four groups based on concordance and discordance between WC and WHR, and their socio-demographic and behavioural profiles were compared, WC identified a group with a more adverse risk profile compared to WHR. For instance, WC-defined groups had a higher prevalence of urban residence, overweight/obesity by BMI, wealth status and parity. The WHR-only group, on the other hand, had a comparatively lower risk profile, which was similar to the profile of the normal population. This suggests that WC may better identify women with clustering of metabolic and lifestyle-related risk factors, whereas WHR may capture a broader and more heterogeneous group.

The association between AO and BMI also differed substantially between the two measures. WC-defined AO showed a steep and progressive increase across BMI categories, rising sharply from underweight to obese women. In contrast, WHR classified a substantial proportion of underweight and normal BMI women as abdominally obese. Kurpad et al. also reported that WC correlated better with BMI than WHR among Asian Indians, suggesting that WC may better reflect overall and abdominal adiposity [25]. They further observed that WHR identified a larger proportion of undernourished and normal-weight women as abdominally obese compared to WC. They also suggested that WHR can be misleadingly low in some obese subjects. Ahmad et al., in a Malaysian population, reported through receiver operating characteristic (ROC) analysis that WC had better sensitivity and specificity than WHR [9]. These findings, along with the present study, are important because BMI has traditionally been used as a measure of generalised obesity. The stronger alignment of WC with BMI may indicate greater consistency in identifying adiposity-related risk.

Another key objective of this study was to examine the association between AO and cardiometabolic outcomes. Our findings showed that both diabetes and hypertension were more common among women with AO compared to non-obese, irrespective of the measure used. The regression analysis was performed to further assess the strength of these associations. After adjusting for socioeconomic and behavioural factors, WC-defined AO showed a stronger association with both diabetes and hypertension compared to WHR-defined AO. These findings are broadly aligned with previous studies.

Wei et al. followed adults aged 25-64 years who were free from diabetes at baseline for more than seven years and found that WC was the strongest predictor of diabetes [26]. The predictive ability of WC alone was at least equal to that of BMI and WHR combined. Similarly, Zhang et al. in China reported that all four anthropometric measures, i.e., BMI, WC, WHR, and waist-to-height ratio (WHtR), were associated with diabetes. But WC and WHtR showed comparatively stronger associations than BMI and WHR [27]. Freemantle et al., in a meta-analysis, also reported a strong overall association between AO and future type 2 diabetes, with a pooled odds ratio of 2.14 [28]. However, the review noted that all measures of AO showed a significant association with diabetes risk. Evidence from South Asia by Patel et al. showed that no single obesity index outperformed the others across all outcomes [29]. Nevertheless, WC and WHtR showed comparatively better performance for identifying diabetes and hypertension, and the authors emphasised the usefulness of WC in cardiovascular disease surveillance among South Asian populations. These studies suggest that AO is consistently associated with diabetes and cardiometabolic risk across populations, but the relative performance of WC and WHR is not identical across populations. Overall, the available evidence, including findings from the present study, suggests that WC may provide a more direct and clinically meaningful measure of abdominal adiposity.

From a policy and programmatic perspective, these findings have clear implications. Population-level screening and surveillance systems require anthropometric measures that are simple, interpretable and feasible for large-scale use. While both WC and WHR are relatively easy to measure, the findings from this study and previous evidence suggest that WC may provide a more direct and clinically meaningful measure of abdominal adiposity and cardiometabolic risk. Global recommendations have also emphasised the inclusion of WC as a routine measure in clinical practice, recognising its value as an important 'vital sign' for assessing metabolic risk [30]. Integrating WC into routine screening, particularly within primary healthcare services, may improve early identification of individuals at risk and support more targeted prevention strategies.

Given the high burden of AO among Indian women, there is also a need to further develop population-specific cut-offs for WC and WHR. The study findings suggest that WHR should be interpreted cautiously. It can be misleadingly high in some cases. In addition, accurate measurement of hip circumference may be more challenging in field settings due to privacy, cultural and operational considerations [25]. This further supports the practical relevance of WC in large-scale public health screening.

This study has several strengths. It uses a large and nationally representative dataset, which allowed robust estimation and subgroup analysis. The study also employed multiple analytical approaches, including discordance analysis, agreement statistics, BMI comparison, and multivariate regression modelling, to provide a comprehensive comparison between WC and WHR. However, certain limitations should also be acknowledged. Anthropometric measurements were taken at a single time point, which may lead to some variation in measurement. In addition, some important behavioural factors, such as physical activity and detailed dietary intake information, were not captured in NFHS-5, which may have resulted in residual confounding. Finally, the cross-sectional nature of the study limits causal interpretation and does not allow assessment of the long-term predictive performance of the two measures for future cardiometabolic risks in these populations.

## Conclusions

There is a high prevalence of AO among Indian women, irrespective of the measure used. WC and WHR identify different groups of women and therefore should not be considered interchangeable measures of AO. WHR identified a substantially larger proportion of women as abdominally obese, particularly among younger and lower-BMI women, whereas WC showed stronger alignment with BMI and stronger association with diabetes and hypertension. These findings suggest that WC may provide a more meaningful measure of AO and more strongly identify women at higher cardiometabolic risk.

The study has important implications for population-level screening and surveillance in India. Selection of an appropriate anthropometric indicator is important because different measures may identify different population subgroups and produce different estimates of the AO burden. Given its simplicity, interpretability and stronger association with cardiometabolic outcomes, WC may be a more suitable measure for routine screening and surveillance. Integrating WC into routine primary healthcare services can improve early identification and timely management of cardiometabolic risk among women in India.
